# Development and characterization of novel microsatellite loci for Lusitanian toadfish, *Halobatrachus didactylus*

**DOI:** 10.7717/peerj.731

**Published:** 2015-01-20

**Authors:** Carla Sousa-Santos, Paulo J. Fonseca, Maria Clara P. Amorim

**Affiliations:** 1 MARE—Marine and Environmental Sciences Centre, ISPA—Instituto Universitário, Rua Jardim do Tabaco, Lisbon, Portugal; 2 Departamento de Biologia Animal and Centro de Biologia Ambiental, Faculdade de Ciências, Universidade de Lisboa, Campo Grande, Lisbon, Portugal

**Keywords:** Batrachoididae, Paternity, Polymorphic loci, Microsatellite development, Pyrosequencing

## Abstract

The Lusitanian toadfish *Halobatrachus didactylus* is an eastern Atlantic polygynous species showing male paternal care. In this paper we describe 5 novel microsatellite loci obtained by 454 GS-FLX Titanium pyrosequencing of a microsatellite-enriched library. The number of alleles per polymorphic locus varied between 2 and 4, and the observed heterozygosity ranged from 0.082 to 0.600. No significant deviation from Hardy–Weinberg equilibrium was found and there was no evidence for linkage disequilibrium. These markers will be of great value for paternity studies and population genetics of this species.

## Introduction

Microsatellite markers (Tautz, 1989), i.e., highly variable DNA sequences of tandem repeats of 1–6 nucleotides with codominant inheritance, are widely used genetic markers in areas as diverse as phylogeography (e.g., Fauvelot & Borsa, 2011), molecular ecology (e.g., Gardner et al., 2011) and parentage analysis (e.g., Jones et al., 2010). Despite their widespread use, the development of species-specific microsatellites can be challenging, especially when a genomic library needs to be constructed and microsatellites must be developed *de novo*. This need arises when there are no microsatellite primers developed for closely related species.

In this work we describe novel microsatellite loci for Lusitanian toadfish *Halobatrachus didactylus* (Bloch & Schneider, 1801) (Batrachoididae), obtained by 454 GS-FLX Titanium pyrosequencing that will be of great value for population genetics studies, namely to conduct paternity studies aiming to clarify the mating dynamics of this species.

The Lusitanian toadfish *H. didactylus* is a benthic fish species usually inhabiting shallow waters down to 50 m depth. It occurs in the eastern Atlantic, from Ghana to the Iberian Peninsula, and has its northernmost distribution limit at the Tagus estuary, in Portugal (Roux, 1986; Bauchot, 1987; Costa, Almeida & Costa, 2003). In the breeding season males of *H. didactylus* defend nests under rocks, where they vocalize to attract females to mate with them (Vasconcelos et al., 2012). Each female spawns a single batch of eggs annually, but males can guard the eggs of several females (Modesto & Canário, 2003; Amorim et al., 2010). In addition to nest-holder (type I) males there is a second male morphotype (type II) that shows morphological and physiological differences from type I males (Modesto & Canário, 2003). Type II males diverge in reproductive tactics and are specialised in sneaking fertilizations (Modesto & Canário, 2003). Interestingly, in the coast of Portugal there are several populations that differ in the degree of incidence of alternative reproductive tactics (ART). While in Tagus estuary type II males are relatively rare (ca. 10%), in Mira estuary they represent 70% of the male population (Pereira et al., 2011). This divergence makes the Lusitanian toadfish an excellent model to study the evolution of mating systems and of ARTs.

## Materials & Methods

Total genomic DNA was isolated from 10 individuals using a *NucleoSpin tissue 96* kit (Macherey-Nagel) following the manufacture’s protocol, except for elution volume (we used 70 µL of elution buffer instead of 100 µL to increase DNA concentration). A total of 1 µg was used for the development of microsatellite libraries through 454 GS-FLX Titanium pyrosequencing of enriched DNA libraries, as described in Malausa et al. (2011). Briefly, total DNA was enriched for AG, AC, AAC, AAG, AGG, ACG, ACAT, and ATCT repeat motifs and subsequently amplified. PCR products were purified and quantified, and GsFLX libraries were then constructed following manufacturer’s protocols (Roche Diagnostics) and sequenced on a GsFLX-PTP. The bioinformatics program QDD (Meglécz et al., 2010) was used to analyse sequences. QDD treats all bioinformatics steps from raw sequences until obtaining PCR primers, including removal of adapters/vectors, detection of microsatellites, detection of redundancy/possible mobile element association, selection of sequences with target microsatellites and primer design by using BLAST, ClustalW and Primer3 programs. Finally, among 10,442 sequences comprising microsatellites motifs, 427 bioinformatic validated pairs of primer were retrieved.

For the validation step, a sub-group of 48 primers pairs was tested for amplification on 14 DNA samples. PCR amplifications were performed in 25 µL reactions containing 20 ng of template DNA, 1×reaction buffer, 37.5 pmol MgCl_2_, 6 pmol dNTP, 10 pmol of each primer, and 1U Taq polymerase (FastStart—Roche Diagnostics). The PCR cycling consisted of an initial denaturation at 95 °C for 10 min, followed by 40 cycles comprising denaturation at 95 °C for 30 s, annealing at 55 °C for 30 s, and extension at 72 °C for 1 min and a final extension at 72 °C for 10 min.

Primer pairs were discarded if they failed to amplify or led to multiple fragments. From the 48 tested primers pairs, 15 were validated. From these, 12 microsatellite loci were selected for polymorphism study on seven DNA samples.

PCR amplifications were performed with the same conditions as previously but with labelled primers. Each PCR product was diluted with dH_2_O (1:50), mixed with Hi-Di Formamide and GeneScan 500 LIZ Size Standard (Applied Biosystems). Fragments were separated using an Applied Biosystems 3730XL DNA Analyser. Alleles were scored using GeneMapper v5.0 software (Applied Biosystems).

Finally, polymorphic markers (sequences available in Table S1 and deposited in GenBank under the accession numbers KP250581–KP250585) were used to optimize multiplexed PCR and afterwards for population genotyping following the above mentioned PCR conditions. The software Multiplex Manager v1.2 was used to check for markers compatibility and to avoid problems like hairpins and primer-dimers. All the laboratorial procedures described above were conducted at GenoScreen-France (www.genoscreen.com).

Conditions and characteristics of the polymorphic loci are provided in Table 1. The number of alleles, expected (*H_e_*) and observed (*H_o_*) heterozygosity (computed using Levene’s correction), and inbreeding coefficients (*F_IS_*) were calculated with GENEPOP on the web (http://genepop.curtin.edu.au) and based on a sample of 85 individuals from the Tagus estuary. Allele sequences are provided in Table S1.

**Table 1:** Characterization of 5 polymorphic microsatellite loci for *H. didactylus*.

Marker	Forward primer sequence (5’–3’)	Reverse primer sequence (5’–3’)	Fluorescent dye	Repeat motif	Expected size (bp)	Size range (bp)	*N_a_*	*H_e_*	*H_o_*	*F_IS_*
Loc11	TCACCTGTGAGAGCGAGAAA	TGCACCTGATCCTAAATCCA	VIC	(GA)_16_	135	121–135	2	0.101	0.106	−0.050
Loc16	ACTCGAACCACAATTCTGCC	CGAACAGGGAAGGAAATCAC	NED	(AC)_18_	110	107–114	4	0.628	0.600	0.045
Loc26	ATGTCTCTTTGTACATTTGTATCTCTG	AAAACTACCAACTGGTCCTCACA	6FAM	(TG)_11_	148	157–159	2	0.322	0.353	−0.097
Loc27	AACATCAGAACATCTGTCAATTCAA	GTCTGGTGCACATGGTGAGT	VIC	(AC)_12_	290	295–297	2	0.079	0.082	−0.038
Loc36	GAAAGGGTCACCATGACAGG	TGCCAACAGTGAAGCAGTTT	PET	(AC)_14_	139	145–167	3	0.452	0.482	−0.067

**Notes.**

Size range of fragments (bp), number of alleles (*N_a_*), expected (*H_e_*) and observed (*H_o_*) heterozygosity, and inbreeding coefficient (*F_IS_*), based on a sample of 85 individuals.

This study was approved by the scientific committee of the Portuguese Foundation for Science and Technology (FCT) which evaluated the project “Role of acoustic signals in mate choice and male–male assessment in a strongly-vocal fish, *Halobatrachus didactylus*” (ref. PTDC/MAR/118767/2010) and the ethical forms filled by the research team. MCP Amorim and PJ Fonseca are also credited by the “Direcção Geral de Alimentação e Veterinária” (General Directorate of Food and Veterinary) as Coordinator-researcher (C category) by FELASA.

## Results and Discussion

The average number of alleles per locus was of 2.6 (Table 1). GENEPOP results showed that there was no heterozygote deficiency (*p*-values between 0.23 and 1.00) and no significant deviation from Hardy–Weinberg equilibrium was found (*X*^2^ = 2.95, *df* = 10, *p* = 0.98; Fisher’s method). The average observed heterozygosity over all loci (Ho) and the level of expected heterozygosity (He) were 0.325 and 0.317, respectively (Table 1). We also tested for the presence of linkage disequilibrium (LD) between pairs of loci using GENEPOP but no evidence for LD was detected (*p*-values ranged from 0.18 to 1.00, mean = 0.62).

Only five of the 12 tested loci were considered polymorphic for this population (polymorphism rate of 41.7%), which is in agreement with the extremely low levels of genetic diversity obtained for this species by other authors (e.g., Marques et al., 2006; Robalo et al., 2013). The fact that our study was focused on a single population of the target species might have also contributed for the low number of polymorphic loci. Nevertheless, the five polymorphic loci were sufficient to address the paternity of eggs (M Amorim, 2014, unpublished data) and so to estimate the impact of sneaking in this population. Moreover, it seems that testing more loci does not necessarily imply a higher detection of polymorphic loci.

**Table 2:** Overview of a brief search on polymorphic microsatellites developed by 454 sequencing for fish species: number of identified, tested and polymorphic loci and their comparison with the values obtain for the present study.

Authors	Target species	Identified loci (IL)	Tested loci (TL)	Polymorphic loci (PL)	TL/IL	PL/TL
	*Halobatrachus didactylus*	10,442	12	5	0%	42%
Cardoso et al., 2013	*Salaria pavo*	4,190	28	26	1%	93%
Carvalho & Beheregaray, 2010	*Conorhynchus conirostris*	3,796	20	13	1%	65%
Quintela et al., 2014	*Labrus bergylta*	92	43	22	47%	51%
Kang, Park & Jo, 2012	*Raja pulchra*	17,033	20	14	0%	70%
Wang et al., 2012	*Megalobrama pellegrini*	24,522	33	26	0%	79%
An et al., 2013	*Stephanolepis cirrhifer*	5,350	74	24	1%	32% ^a^
Zeng et al., 2013	*Acipenser dabryanus*	17,609	80	8	0%	10% ^a^
Carvalho, Hammer & Beheregaray, 2011	*Nannoperca obscura*	9,476	21	15	0%	71%
Umbers et al., 2012	*Gambusia holbrooki*	1,187	40	25	3%	63%
Muths & Bourjea, 2011	*Lutjanus kasmira*	3,022	16	13	1%	81%
Dubut et al., 2010	*Zingel asper*	241	105	55	44%	52%
Dubut et al., 2010	*Sander lucioperca*	241	47	18	20%	38% ^a^
Dubut et al., 2010	*Perca fluviatilis*	241	35	14	15%	40% ^a^
Lü et al., 2013	*Pseudosciaena crocea*	2,535	32	27	1%	84%
Teixeira et al., 2013	*Lepadogaster lepadogaster*	10,258	25	15	0%	60%
	MEAN VALUES	6214.2	41.3	21.0	**9%**	**59%**

**Notes.**

a Studies for which the PL/TL ratio was lower than that obtained in the present study.

To compare the present results with the ones of other studies, we did a brief keyword search for “454 sequencing,” “microsatellite development,” “fish” and “polymorphic.” For the first 15 papers retrieved, we compiled the number of microsatellites detected, the number of tested microsatellites and, from those, the number of polymorphic loci detected. The results, described in Table 2 show that the number of loci tested in our study was below the average (0% vs. 9%) and that the ratio between the number of polymorphic loci (PL) and the number of tested loci (TL) was lower in our study (42%) than the mean for the considered studies (59%). However, a higher number of tested loci did not necessarily imply a higher polymorphism detection (as depicted by the marked (a) PL/TL ratios in Table 2). In addition, although we have detected a low number of polymorphic loci, Neff, Repka & Gross (2000) points out that efforts should only concentrate on increasing the number of loci when the male probable paternity is low. In the studied population the probability of paternity by the nest-holder is high since a preliminary study using these five polymorphic loci shows that nest-holders sire a high percentage of the eggs found in their nests (M Amorim, 2014, unpublished data). These microsatellite markers will allows us to estimate the proportion of eggs sired by the nest-holder and by other males, the number of contributing females to the batch of eggs defended by nest-holders, and the existence of filial cannibalism. They will also contribute to assessing the fitness of different ART in Lusitanian toadfish populations with contrasting incidence of type II males.

## Supplemental Information

10.7717/peerj.731/supp-1 Table S1 Sequences of the five polymorphic microsatellite loci

## Additional Information and Declarations

### Data Deposition

The following information was supplied regarding the deposition of related data: GenBank (accession numbers KP250581–KP250585).
