# Supplementary material for: Development and characterization of novel microsatellite loci for Lusitanian toadfish, Halobatrachus didactylus
Source: PeerJ. 2015 Jan 20;3:e731. doi: 10.7717/peerj.731 (PMC4304857; doi:10.7717/peerj.731)
Supplement: Table S1 [file peerj-03-731-s001.doc]

>*Halobatrachus didactylus* microsatellite locus LOC11

AATGTGAACCGGTTCTACCACTGCTGGATCCGGACCACCATCATCCTCCAGCTCTGCAACCCAGGACGGCACTCCGGTTCTGGAGTCAGGAGGCGGAAGATCACCTGTGAGAGCGAGAAAAagagagagagagagagagagagagagagagagagCTGACAAATAACCTTACTATTAAACTACCAAACTACAGACGAGTCTGGAAAAGACAGACATGGATTTAGGATCAGGTGCAGCTCCGCCTCTGAACTAGTTTCTTTTGTTTCTGTGAACAGTGTGTGATG

>*Halobatrachus didactylus* microsatellite locus LOC16

CCAACACGAGTGTTCGCACTGCAGTGGGAGGGGCTAAAAGCTCGGCGCTGCCGCTAAGCTGCTGTCAATGTGGCTGATCAGTTTTTACAAATCTGTAACGTTGGAACCTTAAAGTCAAACAACGTCATCAGATCACTAAGTGAAGAGCAACCTGACGCCAGCTGGTAACTATTTTCACGCCATTTGGACCCGACGGCCACTTTATTCATGTTCAGGCGCACAAAAAACCCAGAATCCCACTCGAACCACAATTCTGCCGACACGGCGGCATTTCTcacacacacacacacacacacacacacacacacacacaGAGATAAGAAGTAGAGTGATTTCCTTCCCTGTTCGTGCCTCTGAGTCGC

>*Halobatrachus didactylus* microsatellite locus LOC26

TTCCATGCAtttttttttCCCCATCTGTGTCTGTAAtgtgtgtgTTTCTGTTGTGTTTCTGTTGTGTTTCTGTTGTGTTTCTGTTGTGTTTTAGTGCTGTGTGAACTTTTTCATGTCTCTTTGTACATTTGTATCTCTGTATCTATGAGAGGATTGGTTGTGTTCCAGTGCTTCAATGAATATTTATAATTCtgtgtgtgtgtgtgtgtgtgtgTTATTTTATACATACTGTGTCTCTGTGAGGACCAGTTGGTAGTTTTATACCTACTAAAGTGTATTTGCAGAAGT

>*Halobatrachus didactylus* microsatellite locus LOC27

AACATCAGAACATCTGTCAATTCAAGATGAACTGAATGCACTCATCAGGTTAAATGCACGTCTCATTGGATGGAACGGTGATATTTGTCATCTGAACCGTCACCGTCTGTCCGTCCACACCGCCAAACGTCCAGCTGGTGTTTGTGAACGCCCCAAGCTCCTGCTCCTGCAACTGCACCTTACAGCTGATCGATGAGGTGAGCCGGCTCACGTCTGCGcacacacacacacacacacacacaCGTTAACATCATTCACATCATGATATCAACTCACCATGTGCACCAGACTACGAAAAAACTAAATAACTACCCAAAACAGTGCTCTAGTTTGCTGTCTGTNTATGCGTCtgtgtgtgtgtgtgTCTGTGTGT

>*Halobatrachus didactylus* microsatellite locus LOC36

CATGCAGTGTGAAAGGGTCACCATGACAGGGCCCCTGTGTTGTAGCTGACATGCTCCAGTGCAGAGAATacacacacacacacacacacacacacacATGTTGTTGCAGTTCTACTTTAGCATTCTNCAAAACTGCTTCACTGTTGGCATCATTTGTTCTTTTCAGACATGCAAGNCTTTAATGTAATGGACATCTATCTGTACAAAATGNNTTTAATTTTAACTTTCAAACATGTCTCTGTGTTAACAAGTTCAAGTGAACTACACACCGGGGCCCTGTTC
